# A copyright protection scheme for digital images based on shuffled singular value decomposition and visual cryptography

**DOI:** 10.1186/s40064-016-2733-0

**Published:** 2016-07-15

**Authors:** B. Pushpa Devi, Kh. Manglem Singh, Sudipta Roy

**Affiliations:** National Institute of Technology Meghalaya, Shillong, India; National Institute of Technology Manipur, Imphal, India; Assam University, Silchar, India

**Keywords:** Copyright protection, Singular value decomposition, Orthogonal matrix, Visual cryptography, Robust

## Abstract

This paper proposes a new watermarking algorithm based on the shuffled singular value decomposition and the visual cryptography for copyright protection of digital images. It generates the ownership and identification shares of the image based on visual cryptography. It decomposes the image into low and high frequency sub-bands. The low frequency sub-band is further divided into blocks of same size after shuffling it and then the singular value decomposition is applied to each randomly selected block. Shares are generated by comparing one of the elements in the first column of the left orthogonal matrix with its corresponding element in the right orthogonal matrix of the singular value decomposition of the block of the low frequency sub-band. The experimental results show that the proposed scheme clearly verifies the copyright of the digital images, and is robust to withstand several image processing attacks. Comparison with the other related visual cryptography-based algorithms reveals that the proposed method gives better performance. The proposed method is especially resilient against the rotation attack.

## Background

Copyright ownership of multimedia data is vulnerable to the image processing attacks as it can be copied easily without loss of quality with no limitation on the number of copies, tempered and redistributed illegally without authorization due to the growth of the digital multimedia technology tools and omnipresent of the Internet. A good solution to this problem is to integrate the security information directly to the content of digital data in inseparable and/or undetectable form during its useful lifespan (Petticolas 1999), and the digital watermarking is one of such techniques, which embeds the copyright information such as the watermark to the original digital data to be protected without degrading it in an imperceptible manner for the ownership proof purpose in such a way that it is very difficult to detect and remove the information by an unauthorized person. It can be detected or extracted later by the owner to prove his copyright in the case of legal dispute (Hartung and Kutter 1999). Copyright protection of the digital data is defined as the process of proving the intellectual property right to a court of law against unauthorized reproduction, processing, transformation or broadcasting of digital data (Ruanaidh and Pun 1998). For copyright-related applications, the watermarked digital data is expected to be robust to various kinds of geometrical and removal attacks (Cox and Miller 2002).

Classification of the copyright protection of digital document may be based on types of data to be watermarked (text, image, audio or video), working domain being used (spatial or transform), information (blind, semi-blind or non-blind), human perception (visible or invisible), application (source based or destination based), public share (extraction or detection) etc. (Hwang 2000). Some researchers have proposed detection based on the visual cryptography (VC) that does not alter the original image in order to preserve the visual quality of the image, but generates two shares known as the ownership share and the identification share. The ownership share is generated from the original copyrighted document and registered to a certified authority (CA). The identification share is generated from the suspected document. Possessing either one of the shares can not reveal any information related to the copyright, but stacking of two printed shares on transparency sheets conveys meaningful details about the copyright information (Chang et al. 2002; Hsu and Hou 2005; Singh 2009). Visual cryptography based approach has attracted for watermarking of sensitive images such as medical images, where alternation of the pixel values is not permitted (Benyoussef et al. 2015).

Hwang proposed a robust and blind copyright protection scheme based on the visual cryptography that generates shares comparing the most significant bits (MSB) of the pixels in the image with the global mean intensity of the image (Hwang 2000). The probability of false alarm is high in his method. MSB based VC method does not work effectively if the histogram of the grey-level image is either left-skewed or right-skewed (Hassan and Khalili 2005). To overcome this drawback, a blind and robust watermarking scheme for copyright protection of the image in spatial domain using visual cryptography is proposed (Hsu and Hou 2005). It generates the ownership share based on the binary secret message bit, global mean intensity of the image and mean of the neighbouring pixel values of a randomly selected pixels in the image. Their method is robust to many attacks, however it is weak to cropping attack. A slightly different approach to the above two methods is the one that uses the randomly selected pixel value of the image to compare with the global mean intensity for generation of shares (Singh 2009). A robust and blind copyright protection scheme based on the visual cryptography is proposed that generates shares from the product of the normal-distribution random bit and the difference between the low and middle level wavelet sub-bands (Lou et al. 2007). Their method is robust to many attacks, but it was proved that the security of their method depends on the random bit key, but not on the product as the difference between the low wavelet sub-band and the middle wavelet sub-band is always positive (Chen et al. 2009). Abusitta proposed a copyright protection scheme of the image based on the relationship between randomly selected pixels and their 8-neighbours’ pixel and the visual cryptography (Abusitta 2012). His method is an extension of Hwang’s method (Hwang 2000).

A robust and blind watermarking scheme based on visual cryptography is proposed that generates shares comparing the dc coefficient of the discrete cosine transform (DCT) of the block of size 8 × 8 of the image with the mean dc coefficients of blocks from all selected block (Rawat and Raman 2012). Their method is robust to many attacks, but is weak to rotation, cropping, impulse noise, Gaussian noise and sharpening attacks. Jin and Kim proposed an image watermarking scheme based on the DCT and the discrete fractional random transform using the visual cryptography (Jin and Kim 2012). A robust and blind watermarking scheme for copyright protection based on the visual cryptography and the singular values of singular value decomposition (SVD) of the image is proposed that generates shares comparing the mean of the largest singular values from each block in the image with the largest singular value of the selected block (Wang and Chen 2007). The methods mentioned above are robust to many attacks, but it is possible to reveal the secret message using the unauthorized images. Hossaini et al. proposed a robust and blind copyright protection scheme based on the visual cryptography and the steerable pyramid (Hossaini et al. 2016). Their method is robust against against different types of attacks.

A robust watermarking scheme is proposed that embeds the principal component of the watermark of the shuffled SVD (SSVD) of the watermark into the largest singular value of the image block of the host image (Guo and Prasetyo 2014). Right orthogonal matrix is kept as the key for the extraction. False alarm of their method is less. It was reported that the visual quality of the reconstructed image using the SSVD is better than one that uses the SVD.

Motivated by the above discussion, a robust and blind copyright protection algorithm based on the SSVD and VC in the DWT domain is proposed. It decomposes the image into low and high frequency sub-bands and shuffling of pixels is done to the low frequency sub-band. It is then followed by dividing it into similar blocks. Shares are generated based on the difference between one of the elements in the first column of the left orthogonal matrix and its corresponding element in the right orthogonal matrix of the SVD of the blocks in the low frequency sub-band of the image. The experimental results show that the proposed copyright scheme based on the SSVD and the VC is very effective.

The rest of the paper is organized as follows. ‘Preliminaries’ section gives brief preliminaries about the cat map transform, discrete wavelet transform, shuffled singular value decomposition and visual cryptography. ‘Restoration’ section describes the restoration scheme to restore against the rotation, impulse noise and Gaussian noise attacks. ‘Proposed method’ section describes the proposed method. ‘Experimental results’ section gives the experimental results, followed by ‘Conclusions’ in the last section.

## Preliminaries

This section gives a brief overview of the cat map for image pixel shuffling, discrete wavelet transform, singular value decomposition and visual cryptography.

## Cat map

The Arnold cat map is a chaotic bijection of the unit square onto itself, which is used to shuffle coordinates (*x*, *y*) of the image of size *N* × *N*, realizing the effect of image encryption (Fu et al. 2013). The encryption is very slow if the conventional methods of the cryptography for text documents are used, because the size of an image is comparatively big (Wang et al. 2009). The new coordinates (*x*^′^, *y*^′^) on applying the cat map is given as

1 $$ \left[ {\begin{array}{*{20}c} {x^{\prime}} \\ {y^{\prime}} \\ \end{array} } \right] = \left[ {\begin{array}{*{20}c} 1 &\quad a \\ b &\quad  {ab + 1} \\ \end{array} } \right]\left[ {\begin{array}{*{20}c} x \\ y \\ \end{array} } \right]{ \text{mod} }\,N $$

where *a* and *b* are the positive integers, known as the control parameters, and serve as permutation keys.

The cat map is a periodic process, which returns the original position after *P* iterations. This map is area preserving, because the determinant of the transformation matrix is 1. Pixels move with periodicity, and $P, a, b$ and the original image’s side length *N* are correlated; thus, whenever the values $a, b$ and *N* change, it generates a completely different cat map. For shuffling of non-square image of size *M* × *N*, the image is reshaped to a square image with side length $N_{s} = ceil\left( {\sqrt {M \times N} } \right)$, where *ceil*(*x*) returns the value of *x* to the nearest integer greater than or equal to *x* (Fu et al. 2013). The insufficient *N*_*s*_^2^ − *M* × *N* pixels are padded with pseudo random number either 0 or 1 for binary images and in the range 0–255 for other images. Keshavarzian and Aghagolzadeh opine that the cat map provides better security due to the increased number of security keys (Keshavarzian and Aghagolzadeh 2016)

## Discrete wavelet transform

Wavelet is a waveform of limited duration that has an average value of zero, and is used a basal function for representing signals (Mallat 1989). It gives a multiresolution scheme for image representation using different frequencies at different resolution. DWT divides an image of size *M*_1_ × *M*_2_ into four sub-bands $LL, LH, HL$ and *HH*, where *LL* sub-band represents the low frequency of the image and approximation coefficients of DWT, and $LH, HL$ and *HH* indicate the high frequency of the image and are known as the horizontal, vertical and diagonal coefficients respectively. These four sub-bands are approximation, horizontal details, vertical details and diagonal details of the image. One of the next sub-bands can be further processed to obtain the next scale of wavelet coefficients until some final scale is reached.

## Singular value decomposition

The singular value decomposition is an important topic in linear algebra to diagonalize and decompose a matrix into its eigenvectors and eigenvalues (Ranade et al. 2007; Liu and Tan 2002). It has been applied successfully in variety of applications such as data compression, signal processing, pattern analysis, regression analysis, image steganography, watermarking and noise reduction. From the viewpoint of linear algebra, a digital image is a matrix with non-negative elements. SVD of a matrix $H \in {\mathbb{R}}^{M \times N}$ is defined as

2 $$ H = USV^{T} $$

where $U \in {\mathbb{R}}^{M \times M}$ and $V \in {\mathbb{R}}^{N \times N}$ are the left and the right orthogonal matrices such that *UU*^*T*^ = *U*^*T*^*U* = *I*_*M*_ and *VV*^*T*^ = *V*^*T*^*V* = *I*_*N*_, $S \in {\mathbb{R}}^{M \times N}$ is the diagonal matrix consisting of the singular values in a non-increasing order of magnitude and the superscript *T* denotes transpose operator.

It was reported that the performance of the SSVD is better than that of SVD in the reconstructed image quality (Guo and Prasetyo 2014). The SSVD is viewed as a pre-processing of the SVD by permuting the original image with data-independent permutation. The shuffled image $$ \underset{\raise0.3em\hbox{$\smash{\scriptscriptstyle-}$}}{H} $$ of the original image *H* is then fed into the SVD algorithm. The SSVD can be defined as

3 $$ S\left( H \right) = \underset{\raise0.3em\hbox{$\smash{\scriptscriptstyle-}$}}{H}   = \underline{US} \underset{\raise0.3em\hbox{$\smash{\scriptscriptstyle-}$}}{V}^{T} $$

where *S*{.} denotes the shuffling operator. The shuffling operator produces an ensemble image as a low resolution sample of the image.

There are some advantages to employ SVD method in many applications:

1. The size of the block of the image for the SVD transformation is not fixed.
2. The singular value (SV) of the SVD represents the intrinsic algebraic image properties.
3. A small perturbation in the image does not produce large variation in SVs (Wang and Chen 2007).
4. All elements in the first column of the left orthogonal matrix $U$ are of same sign, and differences between them are very small (Su et al. 2013).
5. All elements in the first column of the right orthogonal matrix *V* are of same sign, and differences between them are also very small.
6. A small perturbation in the first column element of the either left or right orthogonal matrix of SVD does not give a large variation in the image.
7. The difference between the corresponding elements in the first column of left and right orthogonal matrices is small.

Such properties can be explored in many copyright protection schemes.

## Visual cryptography

Naor and Shamir introduced visual cryptography in their seminal work in which a secret message is encrypted in a perfectly secure way in more than one shares such that the secret can be decrypted directly by the human visual system (Naor and Shamir 1995). Table 1 illustrates how a binary image of size *N*_*s*_ × *N*_*s*_ is divided into two shares of size 2*N*_*s*_ × 2*N*_*s*_ for a 2-out-of-VC, where each pixel of the secret image is expanded to 2 × 2 subpixels in the shares. If a pixel is white in the secret message, the corresponding subpixels in both two shares are identical, one of six columns under the white pixel in the second and third rows are selected, and the stacked result contains two white subpixels and two black subpixels. On the contrary, if a pixel is black in the secret image, the corresponding subpixels in the first share are complement to those in the same spatial position in the second share, and the stacked result contains four black subpixels. Each block of sub-pixels of size 2 × 2 of the two shares is selected randomly, and so the scheme is secure. Possessing of a single share cannot reveal the secret image. Each block of sub-pixels has six alternative pairs of blocks for both white and black pixel bits.

**Table 1:**
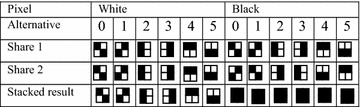
A 2-out-of-2 VC

## Restoration scheme against the rotation, impulse noise and Gaussian noise attacks

The image is passed through a test for the rotation attack. Once the rotation attack is confirmed, the image is then corrected by an image restoration stage. There are two types of rotations—loose and crop. The loose type of the rotation produces an output image large enough to contain the entire rotated image and the crop type produces an output image the same size as the input image, cropping the rotated image to fit.

**Fig. 1:**
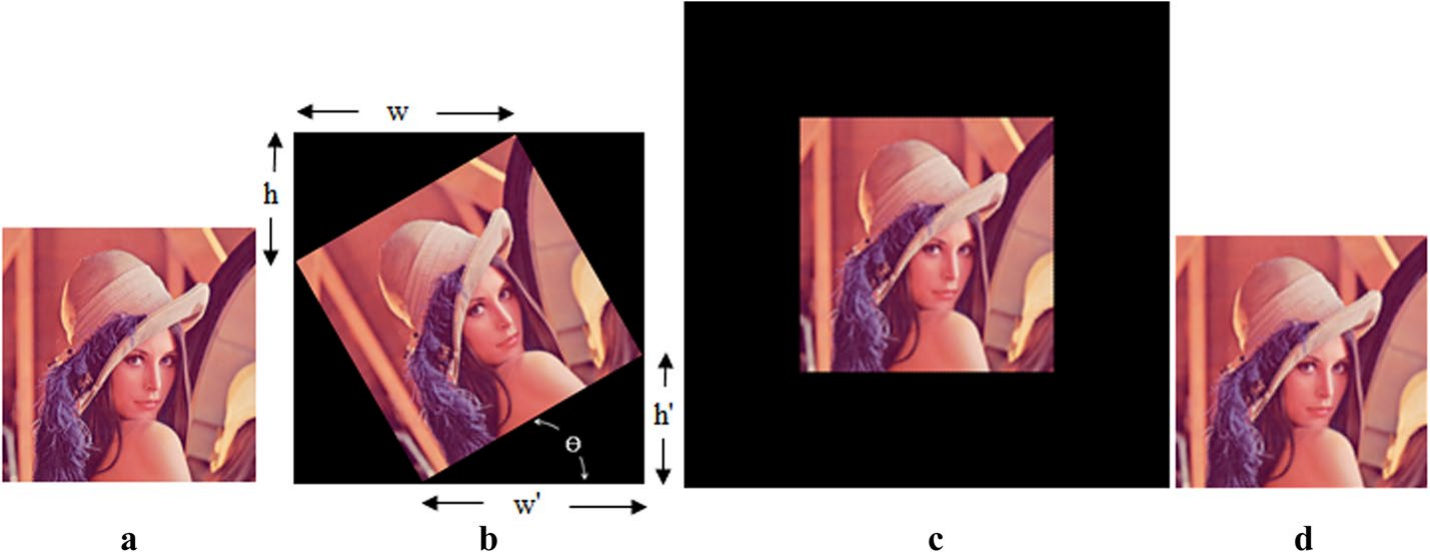
Procedure for the image restoration for the rotation attack **a** Original image, **b** after rotation attack, **c** after restoration and **d** after cropping and scaling

Figure 1 shows the rotation attack and the restoration of the rotated image using the loose type of rotation. The output image after the rotation becomes bigger than the input image and padding with 0 is done wherever necessary due to the increase in size. The rotation is confirmed as shown in Fig. 1b, if *w* = *w*^′^ and *h* = *h*^′^, where $w$ and *w*^′^ are displacements in upper left corner toward right and in bottom right corner towards left, and $h$ and *h*^′^ are displacements in upper left corner toward bottom and in bottom right corner towards top, in the regions with complete dark color. The image can be restored by using the following equation.

4 $$ \left[ {\begin{array}{*{20}c} {x^{\prime}} \\ {y^{\prime}} \\ \end{array} } \right] = \left[ {\begin{array}{*{20}c} {cos\theta } & { - sin\theta } \\ {sin\theta } & {cos\theta } \\ \end{array} } \right]\left[ {\begin{array}{*{20}c} x \\ y \\ \end{array} } \right] $$

where *θ* is the angle of rotation and is found as $\theta = tan^{ - 1} \left( {\frac{\text{w}}{\text{h}}} \right)$, $\left[ {x y} \right]^{T}$ are the coordinates of the pixel value of the rotated image and $\left[ {x^{\prime} y^{\prime}} \right]^{T}$ are coordinates of the pixel value of the corrected image.

The image is rotated by angle of −*θ* in the restoration stage. The size of the corrected image before cropping may be big as shown in Fig. 1c as it is padded with zeros all around. Cropping by removing the padded portion and resizing are done to obtain the final corrected image as shown in Fig. 1d.

A watermarked image may be attacked by the impulse noise and Gaussian noise attacks. The performance of the extraction of the watermark can be improved by smoothing the image with a median filter prior to the extraction (Chang et al. 2014).

## Proposed technique

In this section, the proposed copyright protection scheme is proposed. In order to enhance the security and improve robustness of the proposed watermarking scheme, Arnold cat map is applied to both the image and the watermark (Keshavarzian and Aghagolzadeh 2016). The scheme is divided into two phases: ownership share construction and identification share construction. During the ownership share construction, one of the channels of the color image is used for generation of the share. Padding with the pseudo random number in the appropriate range is done prior to the further processing if the original host image is not square. The selected channel is decomposed first using the DWT into four sub-bands, and the sub-band *LL* is used for generation of the shares. The sub-band *LL* is least effected by any kind of noise suffered by the image (Rani et al. 2015). The ownership share is generated from the *LL* image block of the selected channel of the image by comparing one of the elements in the first column of the left orthogonal matrix with the corresponding element in the right orthogonal matrix of the SSVD of the image block. The share generations based on SSVD–VC in DWT domain are as follows.

## Ownership share generation scheme

**Fig. 2:**
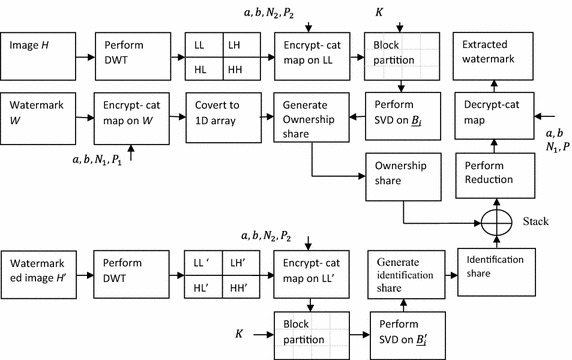
Schematic block diagram of the proposed embedding and extraction scheme

Let *H* be a greyscale image or the selected channel of the host color image of size *M*_1_ × *M*_2_, *W* be the binary watermark of size *N*_*s*_ × *N*_*s*_, *a*${\text{and}} b$ be the control parameters of the cat map for shuffling of pixel coordinates of sub-band *LL* and encrypting the watermark, *P*_2_ and *P*_1_ be the periods of the cat map for the low sub-band *LL* of the image and the watermark respectively, *K* be a private key for selecting the block *B*_*i*_, and *C*_1_ be the codebook as shown in Table 2. Figure 2 shows the schematic diagram of the proposed ownership share generation and identification share generation scheme given. Steps for the ownership share generation are given below.

**Table 2:**
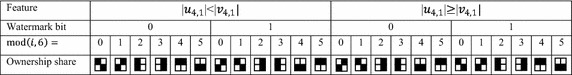
Codebook $\varvec{C}_{1}$ for generation of ownership share

- O1. Perform 1-level DWT on the image *H* of size *M*_1_ × *M*_2_ to obtain four sub-bands $LL, LH, HL$ and *HH*.
  If the image is not square, padding is done using the pseudo random numbers prior to DWT operation to make a square image. Let the size of each sub-band be *M*_*s*_ × *M*_*s*_.
- O2. Apply the cat map on the watermark *W* of size *N*_*s*_ × *N*_*s*_*N*_1_ times using the control parameters *a*, *b* and period *P*_1_ (${\text{where}}\,N_{1} < P_{1} ).$ Store the encrypted watermark in an array.
- O3. Generate a list of random numbers $$ {\text{\{ }}i  | s{\text{uch}} $$$$ {\text{that  total  number  of   random  numbers }} = N_{s} \times N_{s} $$ } using pseudo random number generator (PRNG) with the private key *K*.
- O4. Apply the cat map to the sub-band *LL*, *N*_2_ times using the control parameters *a*, *b* and period *P*_2_ (${\text{where}}\,N_{2} < P_{2} ).$ Then divide the encrypted sub-band into several non-overlapping blocks of size 4 × 4.
- O5. Perform the SVD on a randomly selected block $$ \underset{\raise0.3em\hbox{$\smash{\scriptscriptstyle-}$}}{B}_{i} $$ (*i* denotes the block number) generating the following left orthogonal, singular and right orthogonal matrices.

5 $$ \underset{\raise0.3em\hbox{$\smash{\scriptscriptstyle-}$}}{B}_{i} = \underset{\raise0.3em\hbox{$\smash{\scriptscriptstyle-}$}}{U}_{i} \underset{\raise0.3em\hbox{$\smash{\scriptscriptstyle-}$}}{S}_{i} \underset{\raise0.3em\hbox{$\smash{\scriptscriptstyle-}$}}{V}_{i}^{T} $$

where

$$ \underset{\raise0.3em\hbox{$\smash{\scriptscriptstyle-}$}}{U}_{i} = \left[ {\begin{array}{*{20}c} {u_{1,1} } & {u_{1,2} } & {u_{1,3} } & {u_{1,4} } \\ {u_{2,1} } & {u_{2,2} } & {u_{2,3} } & {u_{2,4} } \\ {u_{3,1} } & {u_{3,2} } & {u_{3,3} } & {u_{3,4} } \\ {u_{4,1} } & {u_{4,2} } & {u_{4,3} } & {u_{4,4} } \\ \end{array} } \right], $$

$$ \underset{\raise0.3em\hbox{$\smash{\scriptscriptstyle-}$}}{S}_{i} = \left[ {\begin{array}{*{20}c} {s_{1,1} } & 0 & 0 & 0 \\ 0 & {s_{2,2} } & 0 & 0 \\ 0 & 0 & {s_{3,3} } & 0 \\ 0 & 0 & 0 & {s_{4,4} } \\ \end{array} } \right] $$

and

$$ \underset{\raise0.3em\hbox{$\smash{\scriptscriptstyle-}$}}{V}_{i} = \left[ {\begin{array}{*{20}c} {v_{1,1} } & {v_{1,2} } & {v_{1,3} } & {v_{1,4} } \\ {v_{2,1} } & {v_{2,2} } & {v_{2,3} } & {v_{2,4} } \\ {v_{3,1} } & {v_{3,2} } & {v_{3,3} } & {v_{3,4} } \\ {v_{4,1} } & {v_{4,2} } & {v_{4,3} } & {v_{4,4} } \\ \end{array} } \right] $$

- O6. Construct the ownership share block *o*_*i*_ based on the feature value $\left( {\left| {u_{4,1} } \right| < \left| {v_{4,1} } \right| \, or \, \left| {u_{4,1} } \right| \ge |v_{4,1} |} \right)$, shuffled watermark bit and ${ \text{mod} }\left( {i,6} \right)$, as shown in the codebook *C*_1_ of Table 2. ${ \text{mod} }\left( {i,6} \right)$ is used to select one of the alternative sub-pixel blocks of the binary bits from the codebook.
- O7. Repeat Steps O5–O6 until all the encrypted watermark bits are exhausted. Finally, all the ownership share blocks are combined to form the ownership share *O*.

After the construction of the ownership share, the watermark, the private key *K*, the control parameters *a*, *b*, the periods *P*_1_, *P*_2_ and the numbers of times of shuffling *N*_1_, *N*_2_ must be kept secretly by the copyright owner, and the ownership share *O* should be registered to a CA for further authentication.

## Identification share generation scheme

Steps of the identification share generation are described below.

- I1. Perform 1-level DWT on the possibly attacked image *H*^′^ of size *M*_1_ × *M*_2_ to obtain four sub-bands $LL^{\prime}, LH^{\prime}, HL^{\prime}$ and *HH*^′^. If the image is not square, padding is done using the pseudo random numbers prior to the DWT operation to make a square image. The size of each sub-band is *M*_*s*_ × *M*_*s*_.
- I2. Generate a list of random numbers $$ {\text{\{ }}i  | s{\text{uch}} $$$$ {\text{that  total  number  of   random  numbers }} = N_{s} \times N_{s} $$ } using pseudo random number generator (PRNG) with the private key *K*.
- I3. Apply the cat map on the sub-band *LL*^′^, *N*_2_ times using the control parameters *a*, *b* and period *P*_2_ (${\text{where}} N_{2} < P_{2} ).$ Then divide the encrypted sub-band into several non-overlapping blocks of size 4 × 4.
- I4. Perform the SVD on a randomly selected block $$ \underset{\raise0.3em\hbox{$\smash{\scriptscriptstyle-}$}}{B}^{\prime}_{i} $$(*i* denotes the block number) generating the left orthogonal, singular and right orthogonal matrices in Eq. 6.

6 $$ \underset{\raise0.3em\hbox{$\smash{\scriptscriptstyle-}$}}{B}^{\prime}_{i} = \underset{\raise0.3em\hbox{$\smash{\scriptscriptstyle-}$}}{U}^{\prime}_{i} \underset{\raise0.3em\hbox{$\smash{\scriptscriptstyle-}$}}{S}^{\prime}_{i} \underset{\raise0.3em\hbox{$\smash{\scriptscriptstyle-}$}}{V}_{i}^{\prime T} $$

where

$$ \underset{\raise0.3em\hbox{$\smash{\scriptscriptstyle-}$}}{U}^{\prime}_{i} = \left[ {\begin{array}{*{20}c} {u_{1,1}^{'} } & {u_{1,2}^{'} } & {u_{1,3}^{'} } & {u_{1,4}^{'} } \\ {u_{2,1}^{'} } & {u_{2,2}^{'} } & {u_{2,3}^{'} } & {u_{2,4}^{'} } \\ {u_{3,1}^{'} } & {u_{3,2}^{'} } & {u_{3,3}^{'} } & {u_{3,4}^{'} } \\ {u_{4,1}^{'} } & {u_{4,2}^{'} } & {u_{4,3}^{'} } & {u_{4,4}^{'} } \\ \end{array} } \right], $$

$$ \underset{\raise0.3em\hbox{$\smash{\scriptscriptstyle-}$}}{S}_{i}^{\prime } = \left[ {\begin{array}{*{20}c} {s_{1,1}^{'} } & 0 & 0 & 0 \\ 0 & {s_{2,2}^{'} } & 0 & 0 \\ 0 & 0 & {s_{3,3}^{'} } & 0 \\ 0 & 0 & 0 & {s_{4,4}^{'} } \\ \end{array} } \right] $$

and

$$ \underset{\raise0.3em\hbox{$\smash{\scriptscriptstyle-}$}}{V}^{\prime}_{i} = \left[ {\begin{array}{*{20}c} {v_{1,1}^{'} } & {v_{1,2}^{'} } & {v_{1,3}^{'} } & {v_{1,4}^{'} } \\ {v_{2,1}^{'} } & {v_{2,2}^{'} } & {v_{2,3}^{'} } & {v_{2,4}^{'} } \\ {v_{3,1}^{'} } & {v_{3,2}^{'} } & {v_{3,3}^{'} } & {v_{3,4}^{'} } \\ {v_{4,1}^{'} } & {v_{4,2}^{'} } & {v_{4,3}^{'} } & {v_{4,4}^{'} } \\ \end{array} } \right] $$

- I5. Construct the identification share block *d*_*i*_ based on the feature value $\left( {\left| {u_{4,1}^{\prime } } \right| < \left| {v_{4,1}^{\prime } } \right| \, or \, \left| {u_{4,1}^{\prime } } \right| \ge \left| {v_{4,1}^{\prime } } \right|} \right)$ and ${ \text{mod} }\left( {i,6} \right)$, as shown in the codebook *C*_2_ of Table 3.

**Table 3:**
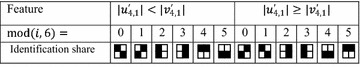
Codebook $\varvec{C}_{2}$ for generation of identification share
- I6. Repeat Steps I4–I5 until all the *N*_*s*_ × *N*_*s*_ blocks are used up from the host image *H*^′^. Finally, all the identification share blocks are combined to form the identification share *D*.
- I7. Retrieve the secret image *W*^′^ of size 2*N*_*s*_ × 2*N*_*s*_ by stacking the ownership share *O* and the identification share *D*.
- I8. Divide the retrieved secret image *W*^′^ into non-overlapping 2 × 2 blocks *s*_*j*,*k*_^′^ (1 ≤ *j*, *k* ≤ 2).
- I9. Perform the reduction process to obtain a reduced secret image *W*^′′^ of size *N*_*s*_ × *N*_*s*_ by the following rules:

7 $$ w = \left\{ {\begin{array}{*{20}c} {1,\quad {\text{if}} \,\mathop \sum \limits_{j} \mathop \sum \limits_{k} s_{j,k}^{'} \ge 2 } \\ {0,\quad {\text{if}}\, \mathop \sum \limits_{j} \mathop \sum \limits_{k} s_{j,k}^{'} < 2} \\ \end{array} } \right. $$

where *w* is a binary pixel in *W*^′′^.

- I10. Scramble the watermark *W*^′′^ by the cat map (*P*_1_ − *N*_1_) times using the control parameters *a* and *b* to obtain the descrambled watermark *W*^′′′^.

## Experimental results

**Fig. 3:**
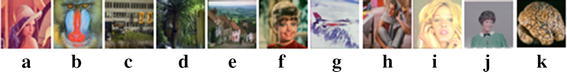
Representative images: **a** Lena, **b** Mandrill, **c** Building, **d** Aptus, **e** Goldhill, **f** Zelda, **g** Airplane, **h** Barbara, **i** Tiffany, **j** Girl and **k** Brain

**Fig. 4:**
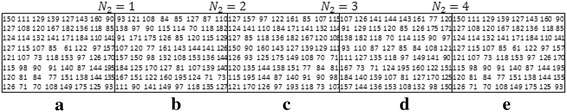
Example of cat map encryption: **a** Pixel values of an image of size 8 × 8, **b** Encrypted pixels with $N_{2} = 1$, **c** Encrypted pixels with $N_{2} = 2$, **d** Encrypted pixels with $N_{2} = 3$ and **e** Encrypted pixels with $N_{2} = 4$

A set of experiments was performed to verify the robustness of the proposed copyright protection algorithm using several images and a binary watermark. Representative images are shown in Fig. 3. The images are Lena, Mandrill, Building, Aptus, Goldhill, Zelda, Airplane, Barbara, Tiffany, Girl and Brain of size 512 × 512 (Sipi, Imagecompression, Cipr, Hlevkin). The original binary watermark of size 64 × 64 is shown in Fig. 4a. The proposed method (PM) is compared with other popular methods in transform domain such as Lou et al. method (LM) (Lou et al. 2007), Wang et al. method (WM) (Wang and Chen 2007) and Rawat et al. method (RM) (Rawat and Raman 2012) that use VC. To evaluate the robustness of the proposed method, the proposed method was tested using ten different types of attacks: JPEG compression (JP), rotation (RO), median filtering (MF), cropping (CR), scaling (SC), impulse noise (IN), blurring (BL), Gaussian noise (GN), sharpening (SH) and Gamma correction (GC). The normalized correlation (NC) is used to measure the similarity between the original watermark and the revealed watermark. It is defined as

8 $$ NC = \frac{{\mathop \sum \nolimits_{m = 1}^{{N_{s} }} \mathop \sum \nolimits_{n = 1}^{{N_{s} }} \overline{{W\left( {m,n} \right) \oplus \overset{\lower0.5em\hbox{$\smash{\scriptscriptstyle\frown}$}}{W} \left( {m,n} \right)}} }}{{N_{s} \times N_{s} }} $$

where *W*(*m*, *n*) and $$ \overset{\lower0.5em\hbox{$\smash{\scriptscriptstyle\frown}$}}{W} \left( {m,n} \right) $$ represent the original secret image and the detected secret image respectively, ⊕ denotes the exclusive-or (XOR) operation and *N*_*s*_ × *N*_*s*_ is the size of the secret image.

PSNR is used to measure the quality of the attacked image. It is given by

9 $$ PSNR = 10{ \log }_{10} \frac{{255^{2} }}{MSE} $$

where MSE stands for mean squared error between the original image and the attacked image.

### Example of cat map encryption

An example of cat map encryption of an image of size 8 × 8 is shown in Fig. 4. Original pixel values are shown in Fig. 4a. Values of control parameters *a* = 3 and *b* = 2 are considered in this example. Figure 4b–e are the encrypted pixel values for *N*_2_ = 1, 2, 3 and 4, where *N*_2_ is the number of iterations. The period *P*_2_ is found to be 4 for these parameters. Figure 4d is the encrypted pixel values after 3 iterations and it should be further encrypted 1 time to get the decrypted image as shown in Fig. 4e. Periods for different combinations of *a* and *b* such as 1 & 1, 1 & 2, 1 & 3, 1 & 4, 2 & 1, 2 & 2, 2 & 3, 2 & 4, 3 & 1, 3 & 2, 3 & 3, 3 & 4, 4 & 1, 4 & 2, 4 & 3 and 4 & 4 are 6, 8, 6,16, 8, 4, 4, 8, 6, 4, 6, 16, 16, 8, 16 and 8 respectively.

The coordinate (0, 0)^′^ having the pixel value of 150 will follow the path shown below for control parameters *a* = 3 and *b* = 2 and image size of 8 × 8 to return to the original position. It is shown below.

$$ \left[ {\begin{array}{*{20}c} 0 \\ 0 \\ \end{array} } \right] \to \left[ {\begin{array}{*{20}c} 4 \\ 1 \\ \end{array} } \right] \to \left[ {\begin{array}{*{20}c} 3 \\ 0 \\ \end{array} } \right] \to \left[ {\begin{array}{*{20}c} 7 \\ 7 \\ \end{array} } \right] \to \left[ {\begin{array}{*{20}c} 0 \\ 0 \\ \end{array} } \right] $$

The original coordinate returns to initial position after 4 iterations. In general, it is claimed that as the value of image size increases, the period tends to increase, but it is not always true (Pages.physics).

### Assessment of robustness

Table 4 shows the robustness test of PM on different types of attacks such as JP attack for quality (Q) from 40 to 90, RO attack for angle (A) of 1°, 2°, 3°, 4°, 5° and 6°, MF attack for window size (ws) of $2 \times 2, 3 \times 3, 4 \times 4, 5 \times 5, 6 \times 6$ and 7 × 7, CR attack for cropping percentage (C) of 10, 20, 30, 40, 50 and 60 %, SC for scaling factor (F) from $2 \times 2, 3 \times 3, 4 \times 4, 5 \times 5, 6 \times 6$ and 7 × 7, IN attack for impulse noise ratio (R) of 0.05, 0.10, 0.15, 0.20, 0.25 and 0.30, GN attack for zero mean and variance (V) from 0.01 to 0.10, BL for sigma (*ζ*) of 0.1, 0.2, 0.3, 0.4, 0.5 and 0.6, SH attack for alpha (*α*) from 0.1 to 1.0 and GC attack for gamma (G) from 0.6 to 1.5. It was found that the performance of PM is very good for JP, RO, MF, SC, IN, BL, GN, SH and GC attacks on different types of images for various ranges and NCs are above 90 % on different values of attacks. The NC values are between 70 and 90 % for CR attack. This shows that PM is robust.

**Table 4:** Robustness test on different images (in NC)

Attack	Lena	Mandrill	Building	Aptus	Goldhill	Zelda	Airplane	Barbara	Tiffany	Girl	Brain
*JPEG Co-mpression*											
Q = 40	97.99	98.58	98.36	97.97	99.34	98.63	97.90	98.24	98.26	98.58	98.73
Q = 50	98.04	98.60	98.77	97.90	99.46	98.70	97.97	98.80	98.43	98.63	98.90
Q = 60	98.24	99.02	98.92	98.55	99.24	98.99	98.33	98.73	98.38	98.80	99.07
Q = 70	98.73	99.36	99.09	98.95	99.48	99.09	98.87	98.95	98.65	98.90	99.12
Q = 80	98.97	99.48	99.26	99.09	99.68	99.38	98.99	99.34	99.02	98.95	99.53
Q = 90	99.31	99.87	99.63	99.63	99.73	99.41	99.34	99.60	99.38	99.43	99.63
*Rotation*											
*A* = 1°	93.82	93.75	94.36	92.50	98.26	96.28	93.31	95.45	92.43	92.55	96.46
*A* = 2°	93.92	93.62	93.23	90.84	97.87	96.31	93.33	95.50	92.40	92.77	96.43
*A* = 3°	95.92	95.33	95.58	93.85	98.21	97.80	95.80	96.24	95.67	96.92	96.41
*A* = 4°	95.80	95.45	95.72	93.55	97.99	97.70	95.77	96.24	95.67	96.99	96.89
*A* = 5°	95.89	95.50	95.62	93.75	97.97	97.66	95.87	96.02	95.77	97.02	96.85
$A = 6^{0}$	96.04	95.45	95.50	93.65	98.26	97.65	95.87	96.31	95.55	97.07	96.45
*Median filter*											
ws = 2 × 2	96.11	95.89	95.97	93.72	98.31	98.02	96.36	95.99	96.48	96.43	96.58
ws = 3 × 3	97.90	96.60	97.77	95.89	99.12	98.87	98.73	97.11	97.43	99.14	98.09
ws = 4 × 4	95.62	94.72	95.23	91.72	98.07	97.58	95.89	95.33	95.31	96.14	96.09
ws = 5 × 5	96.06	94.11	95.23	91.35	98.51	98.07	97.24	95.23	96.31	98.02	96.14
ws = 6 × 6	94.72	93.23	93.62	88.91	97.70	97.16	95.14	94.70	94.84	95.60	95.96
ws = 7 × 7	94.84	92.84	93.89	88.96	98.07	97.48	95.77	94.92	95.62	97.14	94.79
*Cropping*											
%C = 10	86.10	93.03	88.84	89.72	93.79	82.95	80.85	89.86	85.98	79.17	93.77
%C = 20	82.61	89.52	83.76	84.44	89.69	79.68	73.33	83.32	79.10	72.63	87.79
%C = 30	81.56	88.20	79.80	80.15	85.72	77.97	71.48	77.09	77.07	70.77	85.62
%C = 40	78.97	80.17	74.68	78.24	80.54	76.09	71.92	72.46	77.26	70.16	81.32
%C = 50	76.09	77.97	72.36	76.24	76.39	75.46	72.11	71.24	73.48	69.67	78.19
%C = 60	73.33	73.99	69.14	75.46	72.80	74.21	72.85	69.50	74.90	69.67	75.48
*Scaling*											
F = 2 × 2	97.85	96.89	97.80	95.99	99.09	98.99	98.41	97.46	97.92	99.16	98.07
F = 3 × 3	96.16	94.60	95.12	91.82	98.19	98.04	96.67	96.04	96.67	97.63	98.31
F = 4 × 4	94.89	93.43	93.79	89.99	97.58	97.14	95.28	95.45	95.89	96.43	95.14
F = 5 × 5	94.16	92.50	93.28	88.74	97.11	97.04	94.14	94.97	95.04	95.92	93.84
F = 6 × 6	93.55	91.99	92.18	87.35	96.58	96.63	93.28	94.60	94.75	95.28	93.26
F = 7 × 7	92.91	91.87	91.11	86.71	96.14	96.28	92.79	94.21	94.33	95.14	92.57
*Impulse Noise*											
R = .05	97.72	96.41	97.75	95.55	99.09	98.75	98.51	96.53	97.38	99.07	97.80
R = .10	97.41	96.04	97.60	95.84	98.77	98.92	98.16	96.43	97.14	99.02	97.82
R = .15	97.07	95.89	96.85	94.45	98.87	98.33	97.65	96.38	96.77	98.19	97.16
R = .20	96.43	95.21	96.04	93.67	97.99	97.77	96.82	95.31	95.65	97.14	97.11
R = .25	95.43	94.36	95.23	92.26	98.07	96.75	95.89	94.87	94.84	95.99	96.24
R = .30	93.89	93.96	93.82	91.72	97.11	95.87	94.01	94.01	92.89	93.89	95.48
*Blurring*											
*ζ* = 0.1	100	100	100	100	100	100	100	100	100	100	100
*ζ* = 0.2	100	100	100	100	100	100	100	100	100	100	100
*ζ* = 0.3	100	100	99.97	99.97	100	100	100	99.95	99.97	100	99.92
*ζ* = 0.4	99.56	99.53	99.65	99.43	99.82	99.82	99.78	99.65	99.87	99.92	99.65
*ζ* = 0.5	99.29	98.87	99.16	98.60	99.58	99.48	99.34	99.02	99.48	99.63	99.26
*ζ* = 0.6	98.85	98.21	98.70	97.68	99.41	99.38	99.09	98.41	99.36	99.36	98.73
*Gaussian noise*											
V = .01	93.18	95.16	94.77	91.16	97.55	95.77	92.67	95.04	91.94	91.94	96.26
V = .02	91.18	93.89	93.21	89.37	96.82	93.56	90.94	93.75	89.33	88.96	95.31
V = .03	89.81	93.45	91.91	86.93	95.99	92.18	88.59	92.30	87.13	87.40	94.77
V = .04	88.47	92.40	91.82	86.79	95.45	91.11	87.54	91.91	86.27	87.40	93.67
V = .05	87.40	91.89	90.50	86.54	94.75	90.25	86.91	91.77	84.57	85.83	93.01
V = .06	87.08	90.79	90.01	85.67	93.87	89.96	86.32	91.13	84.49	84.93	92.67
V = .07	85.67	91.47	88.74	84.27	93.89	88.84	85.32	90.06	83.05	82.73	91.50
V = .08	85.13	90.33	87.91	84.54	93.96	87.45	84.27	90.11	82.91	82.15	91.33
V = .09	84.93	90.08	87.15	82.44	93.35	87.62	84.03	89.64	80.90	82.27	91.67
V = .10	84.30	89.47	86.86	81.86	92.72	87.81	84.15	89.16	81.20	82.22	90.99
*Sharpening*											
*α* = .1	92.08	92.33	92.79	91.33	96.58	95.70	93.57	92.48	93.11	95.48	94.79
*α* = .2	92.28	92.40	93.04	91.62	96.53	95.72	93.70	92.74	93.11	95.41	94.72
*α* = .3	92.28	92.60	93.23	91.74	96.65	95.80	93.87	92.94	93.13	95.48	94.77
*α* = .4	92.40	92.79	93.21	91.79	96.60	95.70	93.94	93.13	93.16	95.53	94.94
*α* = .5	92.40	92.96	93.28	91.89	96.67	95.70	93.92	93.26	93.23	95.55	95.04
*α* = .6	92.40	92.99	93.31	92.08	96.72	95.80	93.92	93.40	93.31	95.65	95.09
*α* = .7	92.45	93.13	93.35	92.21	96.75	95.75	94.04	93.60	93.45	95.60	95.11
*α* = .8	92.48	93.21	93.38	92.16	96.67	95.82	94.09	93.65	93.43	95.65	95.09
*α* = .9	92.57	93.21	93.45	92.23	96.70	95.80	94.14	93.65	93.57	95.67	95.14
*α* = 1	92.67	93.33	93.55	92.26	96.75	95.77	94.16	93.65	93.53	95.70	95.10
*Gamma correction*											
G = .6	97.60	96.80	97.24	97.46	97.29	95.99	99.16	97.02	99.12	98.75	93.33
G = .7	98.29	97.68	97.92	98.02	98.09	97.21	99.31	98.02	99.24	99.04	97.48
G = .8	98.87	98.36	98.51	98.75	98.75	98.02	99.48	98.65	99.53	99.19	98.36
G = .9	99.41	99.21	99.36	99.56	99.48	98.99	99.75	99.38	99.68	99.60	99.24
G = 1	100	100	100	100	100	100	100	100	100	100	100
G = 1.1	99.07	99.31	99.21	99.41	99.43	98.99	99.68	99.36	99.78	99.63	99.29
G = 1.2	98.46	98.73	98.63	98.87	98.87	98.02	99.60	98.85	99.73	99.56	98.75
G = 1.3	98.02	97.97	97.99	98.29	98.43	97.38	99.31	97.94	99.43	99.34	98.09
G = 1.4	97.33	97.41	97.53	97.92	98.09	96.65	99.21	97.50	99.16	99.12	97.43
G = 1.5	96.94	96.99	96.89	97.11	97.87	96.02	98.82	96.94	98.92	99.07	96.87

## Comparison with other methods

**Fig. 5:**
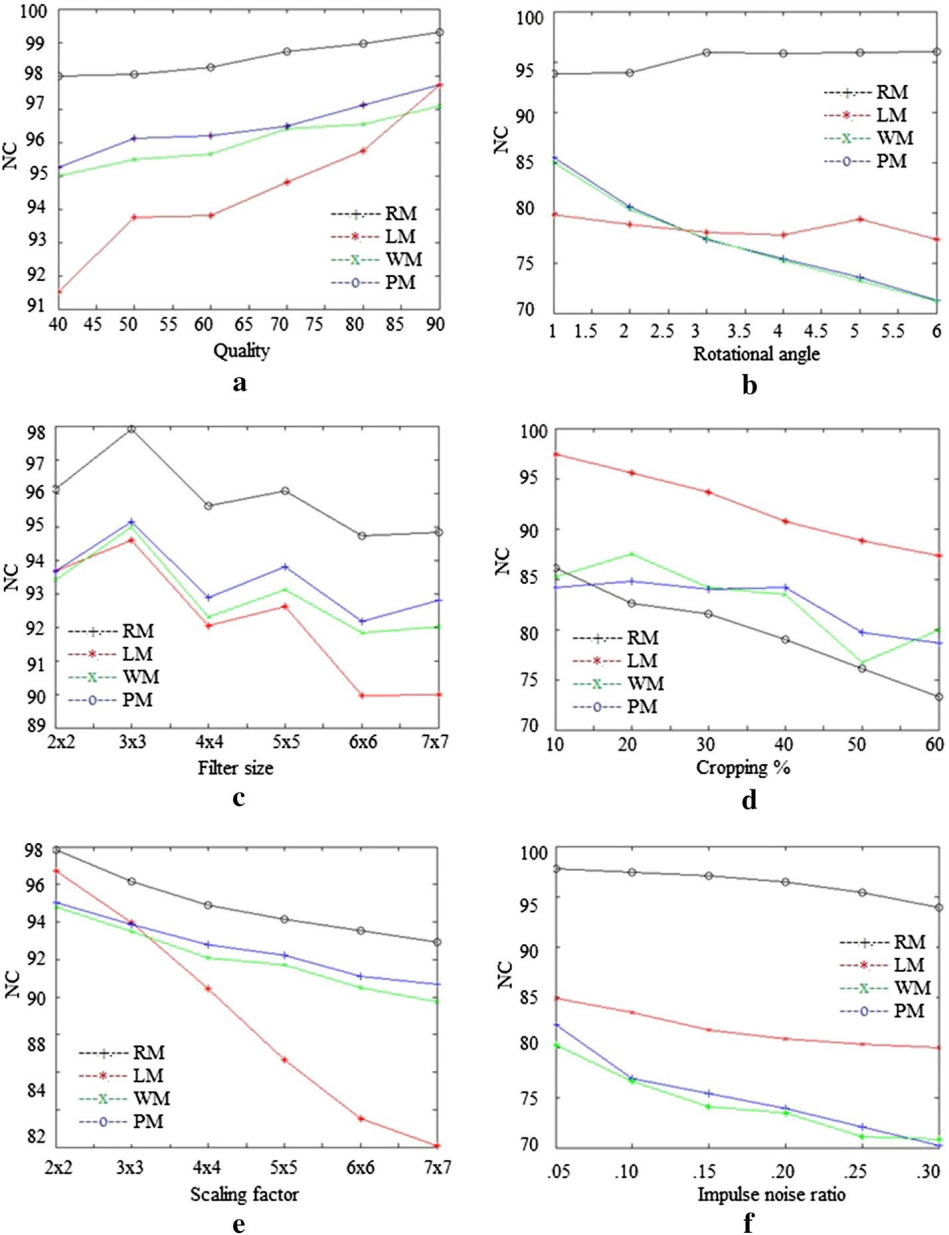
Comparison of different methods: **a** JPEG compression, **b** Rotation, **c** Median filter, **d** Cropping, **e** Scaling, **f** Impulse noise

**Fig. 6:**
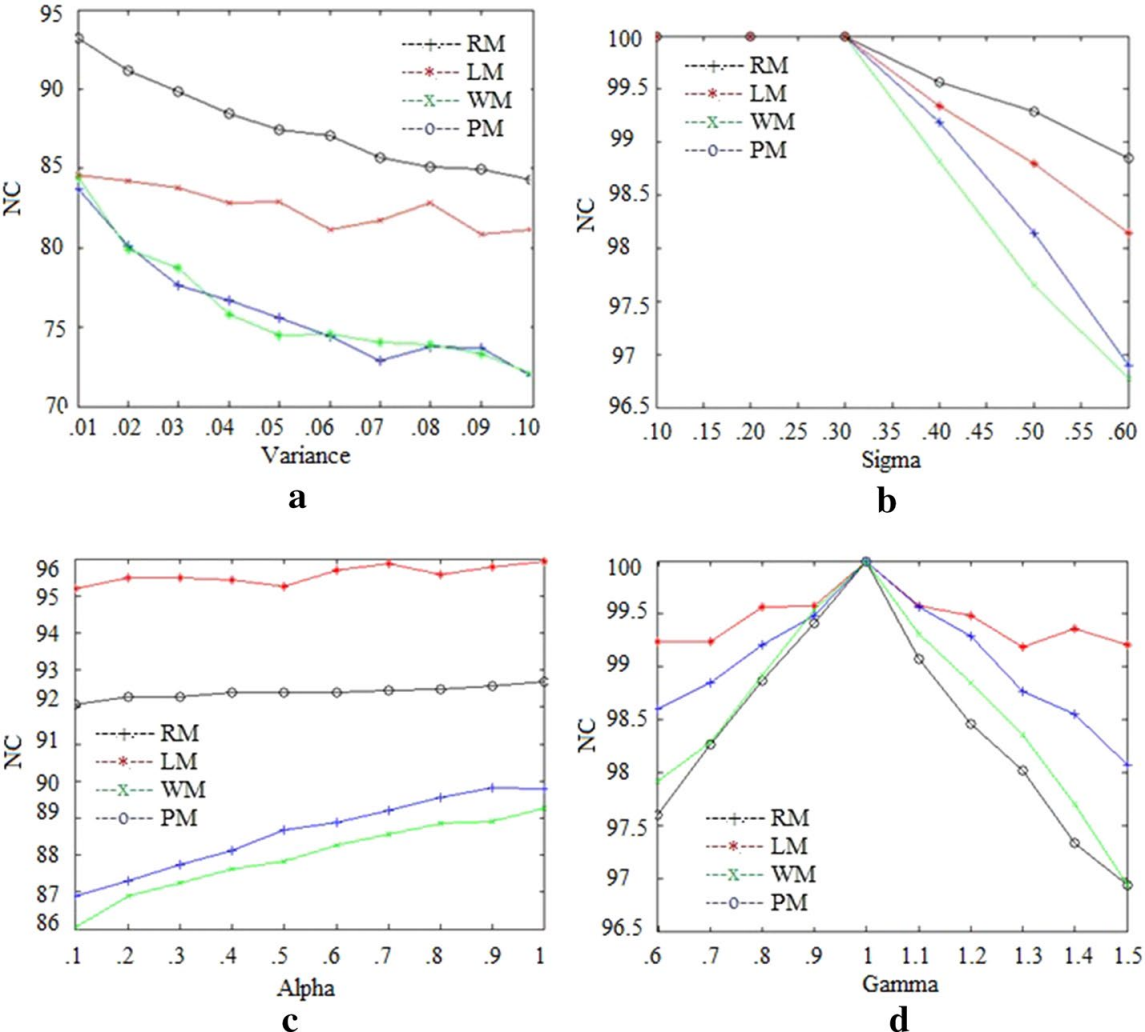
Comparison of different methods: **a** Gaussian noise, **b** Blurring, **c** Sharpening and **d** Gamma correction attacks

Table 5 shows comparison of the proposed method with LM, WM and RM on six different images for ten different attacks. The unweighted average (UA) is also shown in the table. The table clearly shows the superiority of PM to LM, WM and RM in term of NC for JP, RO, MF, SC, IN, BL and GN attacks on different images. LM gives slightly better results for CR, SH and GC attacks. Figure 5 is the graphical comparison of PM, LM, WM and RM for ten attacks on Lena image. The unweighted average in Table 5 and Figs. 5, 6 show the superior performance of PM on different images for different parameters.

**Table 5:** Comparison with other methods

Attack	LM						WM					
	Lena	Building	Goldhill	Airplane	Tiffany	Brain	Lena	Building	Goldhill	Airplane	Tiffany	Brain
*JP*												
Q = 50	93.75	95.84	94.43	92.18	91.94	95.04	95.50	95.45	95.87	96.28	93.50	97.38
Q = 70	94.79	97.41	96.60	93.65	94.53	95.87	96.41	96.36	96.33	97.09	94.70	97.87
Q = 90	97.72	98.75	98.38	96.60	97.14	97.70	97.09	98.24	98.14	98.26	96.77	98.73
*RO*												
*A* = 1°	79.78	79.19	80.46	79.98	81.49	83.96	84.93	79.29	84.79	79.54	79.12	84.61
*A* = 3°	77.29	78.32	78.83	78.54	77.95	78.63	77.39	73.19	73.16	73.31	69.75	75.10
*A* = 5°	79.37	77.73	78.63	78.34	77.46	78.41	73.19	67.04	68.60	70.50	67.43	70.41
*MF*												
ws = 3 × 3	94.60	95.94	95.58	95.89	94.23	97.11	94.99	94.06	94.82	96.09	91.25	94.75
ws = 5 × 5	92.62	92.57	93.11	93.43	91.57	94.11	93.11	90.28	92.99	94.04	88.54	91.77
ws = 7 × 7	89.99	87.93	89.69	90.01	88.35	90.52	92.01	88.35	92.13	91.87	87.54	89.99
*CR*												
%C = 20	95.53	94.45	94.84	95.31	95.62	95.01	87.52	80.88	79.49	72.72	63.62	83.66
%C = 40	90.77	90.62	90.16	91.08	91.45	91.18	83.44	78.44	77.90	72.70	61.23	82.15
%C = 60	87.32	86.32	86.10	86.74	86.69	86.96	80.00	73.53	72.72	70.06	72.97	77.41
*SC*												
F = 2 × 2	96.70	97.75	96.92	95.99	96.33	97.29	94.79	92.94	94.65	94.43	91.16	94.31
F = 4 × 4	90.45	90.40	89.52	88.76	88.89	90.42	92.08	88.59	91.94	91.28	88.08	90.84
F = 6 × 6	83.52	84.35	83.66	83.08	83.49	84.98	90.50	85.76	90.03	88.84	87.06	87.86
*IN*												
R = .05	84.91	86.49	85.79	84.35	83.32	96.70	80.22	79.49	79.32	79.10	76.90	94.53
R = .15	81.71	83.10	83.17	82.00	80.78	94.79	74.04	73.77	75.19	74.38	72.14	93.31
R = .25	80.27	82.34	81.25	81.03	80.46	91.74	71.14	70.26	70.99	71.24	70.31	90.89
*BL*												
*ζ* = 0.2	100	100	100	100	100	100	100	100	100	100	100	100
*ζ* = 0.4	99.34	99.56	99.31	99.14	99.21	99.80	98.82	99.16	99.19	99.29	98.68	99.19
*ζ* = 0.6	98.14	98.75	98.43	98.14	97.97	99.12	96.77	96.46	96.67	97.26	95.33	97.19
*GN*												
V = .01	84.57	87.59	85.96	84.25	83.66	89.28	84.42	84.91	85.15	82.71	78.93	92.60
V = .03	83.25	84.13	83.22	83.78	80.68	87.57	78.73	79.51	79.29	77.49	73.46	89.18
V = .05	82.93	83.83	81.90	82.03	80.90	86.76	74.53	76.34	75.75	75.09	72.09	87.89
SH												
*α* = .1	95.19	95.67	95.99	95.33	94.99	96.41	86.05	87.15	86.81	89.74	84.35	89.52
*α* = .3	95.50	95.72	96.26	95.26	95.38	96.58	87.23	87.81	87.52	90.16	85.64	90.06
*α* = .5	95.28	95.87	96.41	95.53	95.58	96.75	87.84	88.01	87.67	90.60	86.27	90.23
GC												
G = .8	99.56	99.29	99.12	99.02	99.16	99.38	98.92	97.53	98.16	99.21	98.92	97.65
G = 1	100	100	100	100	100	100	100	100	100	100	100	100
G = 1.2	99.48	99.16	99.29	99.29	99.21	98.73	98.85	97.87	98.02	99.34	99.07	97.75
UA	90.81	91.30	91.10	90.62	90.28	93.02	88.35	86.68	87.44	87.08	84.16	90.89

	RM						PM					
*JP*												
Q = 50	96.11	95.31	95.92	96.31	94.14	96.63	98.04	98.77	99.46	97.97	98.43	98.90
Q = 70	96.48	96.75	96.67	96.97	94.89	97.43	98.73	99.09	99.48	98.87	98.65	99.12
Q = 90	97.72	97.92	97.65	98.14	96.80	98.58	99.31	99.63	99.73	99.34	99.38	99.63
*RO*												
*A* = 1°	85.49	78.88	84.93	79.27	77.49	84.83	93.82	94.36	98.26	93.31	92.43	96.46
*A* = 3°	77.34	72.26	73.77	72.46	68.48	76.41	95.92	95.58	98.21	95.80	95.67	96.41
*A* = 5°	73.53	66.62	69.92	70.21	66.47	70.31	95.89	95.62	97.97	95.87	95.77	96.85
*MF*												
ws = 3 × 3	95.14	93.75	94.99	96.38	91.62	94.97	97.90	97.77	99.12	98.73	97.43	98.09
ws = 5 × 5	93.79	90.57	92.99	94.21	88.79	92.30	96.06	95.23	98.51	97.24	96.31	96.14
ws = 7 × 7	92.79	88.89	91.87	92.50	87.25	90.42	94.84	93.89	98.07	95.77	95.62	94.70
*CR*												
%C = 20	84.81	79.41	78.14	72.43	64.74	83.10	82.61	83.76	89.69	73.33	79.10	87.79
%C = 40	84.20	79.24	80.49	74.58	62.67	85.18	78.97	74.68	80.54	71.92	76.26	81.32
%C = 60	78.63	72.80	71.77	68.60	70.09	75.34	73.33	69.14	72.80	72.85	74.90	75.48
*SC*												
F = 2 × 2	95.04	92.55	94.67	94.48	91.74	94.26	97.85	97.80	99.09	98.41	97.92	98.07
F = 4 × 4	92.77	88.23	92.11	90.91	88.79	90.60	94.89	93.79	97.58	95.28	95.89	95.14
F = 6 × 6	91.08	85.40	90.42	88.67	86.96	88.57	93.55	92.18	96.58	93.28	94.75	93.26
*IN*												
R = .05	82.25	81.05	82.78	79.00	76.53	94.92	97.72	97.75	95.55	98.75	97.38	97.80
R = .15	75.36	76.00	76.17	73.65	72.90	93.67	97.07	96.85	94.45	98.33	96.77	97.16
R = .25	72.09	72.38	72.41	70.89	69.77	91.47	95.43	95.23	92.26	96.75	94.84	96.24
*BL*												
*ζ* = 0.2	100	100	100	100	100	100	100	100	100	100	100	100
*ζ* = 0.4	99.19	99.19	99.26	99.41	98.97	99.02	99.56	99.65	99.82	99.78	99.87	99.65
*ζ* = 0.6	96.89	96.36	96.92	97.43	95.72	96.80	98.85	98.70	99.41	99.09	99.36	98.73
*GN*												
V = .01	83.74	84.91	85.64	83.20	77.90	92.30	93.18	94.77	97.55	92.67	91.94	96.26
V = .03	77.61	78.78	79.02	76.63	73.02	89.91	89.81	91.91	95.99	88.59	87.13	94.77
V = .05	75.56	76.12	77.51	76.02	71.87	87.40	87.40	90.50	94.75	86.91	84.57	93.01
*SH*												
*α* = .1	86.88	88.03	87.37	89.06	84.17	90.45	92.08	92.79	96.58	93.57	93.11	94.79
*α* = .3	87.74	88.40	87.79	89.79	85.74	91.04	92.28	93.23	96.65	93.87	93.13	94.77
*α* = .5	88.69	88.62	88.13	90.35	86.79	90.89	92.40	93.28	96.67	93.92	93.23	95.04
*GC*												
G = .8	99.21	98.21	98.46	99.26	98.90	97.80	98.87	98.51	98.75	99.48	99.53	98.36
G = 1	100	100	100	100	100	100	100	100	100	100	100	100
G = 1.2	99.29	98.36	98.60	99.36	99.09	98.16	98.46	98.63	98.87	99.60	99.73	98.75
UA	88.64	86.83	87.87	87.00	84.07	91.09	94.16	94.10	96.07	93.97	93.97	95.42

### No attack

**Fig. 7:**
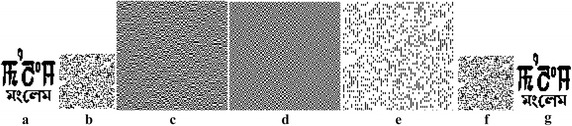
No attack: **a** Original watermark, **b** Encrypted watermark, **c** Ownership share, **d** Identification share, **e** Superimposed share, **f** Superimposed share after reduction and **g** Extracted watermark

Figure 7 shows the various steps to establish the ownership of the copyright. The original binary watermark is encrypted by using the cat map and is shown in Fig. 7b. The ownership share is prepared based on the encrypted watermark and the original image and it is shown in Fig. 7c. The identification share is prepared from the watermarked image and is shown in Fig. 6d. The superimposed image of the ownership share and the identification share is shown in Fig. 7e. It is blurred and not recognizable. The reduction superimposed image before the decryption is shown in Fig. 7f. It is blurred and not recognizable. Figure 7g shows the decrypted watermark, which is exactly similar to the original watermark. NC value of the extracted is 100 and PSNR value of the watermarked image is infinite for no attack.

### JPEG compression attack

**Fig. 8:**
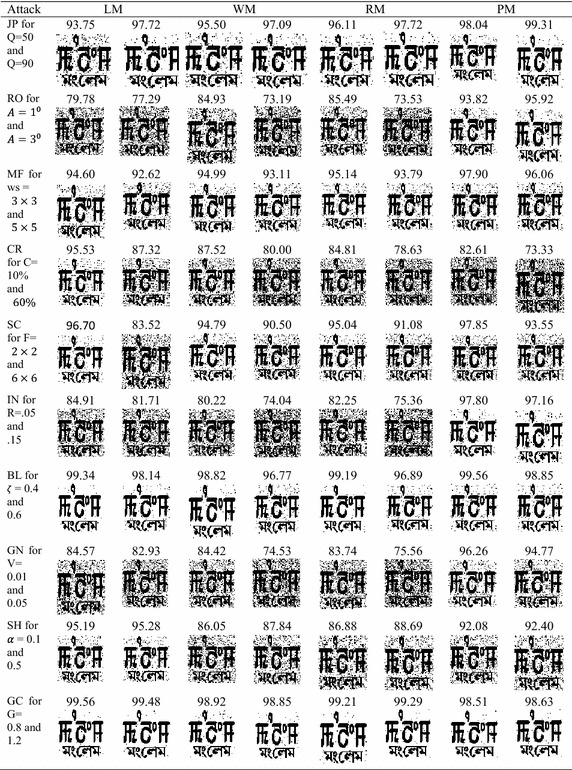
Detected watermarks by various methods

Figure 8 shows the quality of the extracted watermark. NC values of LM, WM, RM and PM for the JPEG compression attack for Q of 90 are 97.72, 97.09, 97.72 and 99.31 respectively with PSNR value of 39.48 dB on Lena image. NC values are 93.75, 95.50, 96.11 and 98.04 respectively with PSNR value of 33.93 dB for LM, WM, RM and PM on Lena image for Q of 50. It clearly shows the superior performance for the JPEG compression attack.

### Rotation attack

Figure 8 shows the quality of the extracted watermark. NC values of LM, WM, RM and PM for the rotation attack for an angle of an 1° are 79.78, 84.93, 85.49 and 93.82 respectively with PSNR values of 21.01 dB for LM, WM and RM respectively and 25.09 dB for PM on Lena image. NC values are 77.29, 73.19, 73.53 and 95.92 respectively with PSNR value of 16.38 dB for LM, WM and RM and 29.29 dB for PM on Lena image for an angle of 3°. It clearly shows the superior performance for the rotation attack.

### Median filter attack

Figure 8 shows the quality of the extracted watermark. NC values of LM, WM, RM and PM for the median filter attack for window size of 3 × 3 are 94.60, 94.99, 95.14 and 97.90 respectively with PSNR value of 36.88 dB on Lena image. NC values are 92.62, 93.11, 93.79 and 96.06 respectively with PSNR values of 34.34 for LM, WM, RM and PM on Lena image for window size of 5 × 5. It clearly shows the superior performance for the median filter attack.

### Cropping attack

The performance of LM is better than the other methods for the cropping attack. However WM, RM and PM also give good and recognizable extracted watermark.

### Scaling attack

NC values of LM, WM, RM and PM for the scaling attack for scale factors of 2 × 2 and 6 × 6 are 96.70, 94.79, 95.04, 98.07 respectively, and 83.52, 90.50, 91.08 and 93.26 respectively with PSNR values 32.99 dB and 26.65 dB. It clearly shows the superior performance for the scaling attack.

### Impulse noise attack

Figure 8 shows the quality of the extracted watermark. NC values of LM, WM, RM and PM for impulse noise attack for impulse noise ratio of 0.05 are 84.91, 80.22, 82.25 and 97.80 respectively with PSNR values of 23.34 dB for LM, WM and RM and 36.42 dB for PM, and for impulse noise ratio of 0.15 are 81.71, 74.04, 75.36 and 97.16 respectively with PSNR values of 18.60 dB for LM, WM and RM and 34.81 dB for PM. It clearly shows the better performance of the proposed method for impulse noise attack. The better performance of PM is due to inbuilt restoration scheme against the impulse noise attack.

### Blurring attack

Figure 8 shows the quality of the extracted watermark. LM, WM, RM and PM give very good performance for blurring attack, and the detected secret images are also not blurred.

### Gaussian noise attack

Figure 8 shows the quality of the extracted watermark. NC values of LM, WM, RM and PM for the Guassian noise attack for zero mean and variance of 0.01 are 84.57, 84.42, 83.74 and 96.26 respectively with PSNR values of 24.77 dB for LM, WM and RM and 30.89 dB for PM, and for variance of 0.03 are 82.93, 74.53, 75.56 and 94.77 respectively with PSNR values of 20.21 dB for LM, WM and RM and 27.08 dB for PM. It clearly shows the better performance of the proposed method for Gaussian noise attack. The better performance of PM is due to inbuilt restoration scheme against the Gaussian noise attack.

### Sharpening attack

Figure 8 shows the quality of the extracted watermark. LM shows better performance in comparison with WM, RM and PM for sharpening attack.

### Gamma correction attack

For gamma correction attack, all LM, WM, RM and PM give good performance.

False positive detection problem arises in most of the SVD and VC-based algorithms. Methods proposed by Lou et al. (2007), Rawat and Raman (2012) and Wang and Chen (2007) suffer from this problem. An unauthorized image can be used to extract or detect the watermark producing the watermark, though the quality is not good. This means that anyone who can detect watermark can claim ownership. Our method solves this false claim by encrypting the watermark prior to the ownership share generation, and it decrypts at the time of detection.

### Robustness against the rotation attack

Table 6 gives the NCs and errors in detection for the rotation angles from 5° to 85° on Lena and Mandrill images. It shows that the NC values of the proposed algorithm for both images lie above 93.67 and below 95. Errors in detection for the rotation angles are comparatively low. The minimum error in magnitude is 0.20 % and the maximum error in magnitude is 3.02 %. It shows that the proposed method is very effective to handle the rotation attack.

**Table 6:** NC values and  % errors of detection of angles for the rotation attack

Lena																	
Rotation	5°	10°	15°	20°	25°	30°	35°	40°	45°	50°	55°	60°	65°	70°	75°	80°	85°
Detection	5.15°	10.17°	15.09°	20.10°	25.13°	30.06°	35.11°	39.99°	45.00°	50.00°	54.88°	59.93°	64.86°	69.86°	74.90°	79.82°	84.84°
Error %	−3.02	−1.78	−0.62	−0.50	−0.53	−0.22	−0.32	0.01	0	0	0.20	0.11	0.20	0.19	0.12	0.22	0.17
NC	95.89	95.84	95.89	95.94	93.67	95.97	95.99	95.80	95.80	95.94	95.89	95.99	95.06	95.92	95.94	95.89	95.84
Mandrill																	
Rotation	5°	10°	15°	20°	25°	30°	35°	40°	45°	50°	55°	60°	65°	70°	75°	80°	85°
Detection	5.11°	10.17°	15.09°	20.10°	25.13°	30.06°	35.11°	39.99°	45°	50°	54.88°	59.93°	64.86°	69.69°	74.90°	79.82°	84.84°
Error %	−3.02	−1.78	−0.62	−0.50	−0.53	−0.22	−0.32	0.01	0	0	0.20	0.11	0.20	0.14	0.12	0.22	0.17
NC	95.50	95.50	95.55	95.48	93.70	93.82	95.43	95.38	93.82	93.67	95.45	95.41	93.79	95.45	95.50	95.48	95.48

## Conclusions

The paper describes a new watermarking algorithm based on the shuffled singular value decomposition and the visual cryptography for copyright protection of digital images in the DWT domain. The robustness of the proposed method was verified on different types of images for different attacks. Comparison with the other related VC-based algorithms reveals that the proposed method gives better performance.
